# Worry, Perceived Threat and Media Communication as Predictors of Self-Protective Behaviors During the COVID-19 Outbreak in Europe

**DOI:** 10.3389/fpsyg.2021.577992

**Published:** 2021-02-16

**Authors:** Martina Vacondio, Giulia Priolo, Stephan Dickert, Nicolao Bonini

**Affiliations:** ^1^Department of Psychology, University of Klagenfurt, Klagenfurt, Austria; ^2^Department of Psychology and Cognitive Sciences, University of Trento, Rovereto, Italy; ^3^Department of Marketing, School of Business and Management, Queen Mary University of London, London, United Kingdom; ^4^Department of Economics and Management, University of Trento, Trento, Italy

**Keywords:** risk perception, precautionary behaviors, coronavirus outbreak, pandemic, COVID-19, framing, emotions

## Abstract

Efforts to contain the spread of the coronavirus emphasize the central role of citizens’ compliance with self-protective behaviors. Understanding the processes underlying the decision to self-protect is, therefore, essential for effective risk communication during the COVID-19 pandemic. In the present study, we investigate the determinants of perceived threat and engagement in self-protective measures in the United Kingdom, Italy, and Austria during the first wave of the pandemic. The type of disease (coronavirus vs. seasonal flu) and the type of numerical information regarding the disease (number of recovered vs. number of dead) were manipulated. Participants’ cognitive and emotional risk assessment as well as self-reported engagement in protective behaviors were measured. Results show that worry was the best predictor of perceived threat in all countries. Moreover, a path analysis revealed that worry and perceived threat serially mediated the effect of type of disease on engagement in self-protective behaviors. The numerical framing manipulation did not significantly impact behavior but had a direct effect on worry and an indirect effect on perceived threat. These results are in line with theoretical accounts that identify emotions as a central determinant for risk perception. Moreover, our findings also suggest that effective risk communication during the COVID-19 pandemic should not stress comparisons to other, well-known viral diseases, as this can ultimately reduce self-protective behaviors.

## Introduction

At the end of 2019, a new coronavirus, known as SARS-CoV-2, rapidly spread from Wuhan, China to the rest of the world, causing the most significant health emergency in recent history. In the absence of a vaccine and effective cures, governments had to rely on non-pharmaceutical (i.e., behavioral) interventions to “flatten the curve” of infections.

Unprecedented public policies (e.g., nationwide lockdowns, travel restriction, social distancing) and preventive behaviors (e.g., wearing a face mask, frequent handwashing with soap) have been stressed by the (World Health Organization, 2020) and were implemented to varying degrees by governments to combat the pandemic. However, the effectiveness of these measures is higher when policies and behaviors are adopted in combination, are implemented promptly, and when citizens’ adherence is nearly universal (Eikenberry et al., 2020; Hsiang et al., 2020; Stutt et al., 2020).

Understanding the drivers of preventive behaviors is, therefore, paramount to boost compliance and increase the effectiveness of containment measures through adequate health campaigns. The general aim of our study is to investigate how emotional reactions and perceived threat influence engagement in self-protective behaviors during the COVID-19 pandemic and if these factors can be affected by media communication content (e.g., information about the type for the disease and the number of affected people). Our study was inspired by the media communication during the early stages of the pandemic, which often highlighted the comparison of the coronavirus to the seasonal flu and was selective in which numbers were presented to describe the pandemic (e.g., initially only the number of affected as well as the number of dead were presented, but not the number of recovered).

### Perceived Threat and Preventive Behaviors

The literature in the health-risk domain considers the subjective perception of a threat to be a major driver of people’s preventive actions. Models such as the Health Beliefs Model (HBM; Hochbaum et al., 1952; Rosenstock, 1960, 1974) and Protection Motivation Theory (PMT; Rogers, 1975; Prentice-Dunn and Rogers, 1986; Rogers and Prentice-Dunn, 1997) include threat perception as a key factor in motivating people toward preventive behaviors. Specifically, the perception of a threat is positively related to people’s intention to undertake protective actions (Brewer et al., 2007; Sheeran et al., 2014). Studies on previous infectious disease outbreaks such as SARS, swine flu, and MERS show a direct association between perceived threat and adherence to mitigating measures (de Zwart et al., 2009; Leppin and Aro, 2009; Rubin et al., 2009; Kim and Song, 2017). Following these theoretical approaches and previous studies, in our research, we define “perceived threat” as the multiplication of two dimensions: the perceived likelihood of contracting a disease (i.e., vulnerability to a hazard) and the perceived severity of it (i.e., perceived negative consequences of a hazard). Consistent with the literature, we expect to find that higher perceived threat will be associated with higher engagement in self-protective behaviors (H_1_).

### Emotional Reactions

A possible limitation of the HBM and PMT models is that they do not adequately account for the role of emotions in perception of threat and risk judgments (Leppin and Aro, 2009). This underestimation of affective reactions can explain the modest associations found between perceived threat and behaviors (Leppin and Aro, 2009; Sheeran et al., 2014). According to frameworks such as the dual-process models (Kahneman and Frederick, 2002; Evans, 2008) and the “*risk as feelings*” approach (Slovic et al., 2004), feelings and emotions can have a predominant role in guiding information processing underlying the perception of risk and benefits (Finucane et al., 2000; Lerner and Keltner, 2000; Loewenstein et al., 2001; Slovic et al., 2002, 2004; Lerner et al., 2003; Peters et al., 2006b; Slovic and Peters, 2006; Sjöberg, 2007; Vacondio and Dickert, 2020). According to this view, emotional reactions come prior to and can direct risk judgments and behavioral reactions.

The role of emotions could be even more prevalent in a highly threatening situation, such as the coronavirus pandemic, due to the lack of clear and precise information (Leppin and Aro, 2009). Indeed, studies on previous pandemics have shown that negative emotions (e.g., worry, anxiety) are correlated with preventive behaviors (Brug et al., 2004; Rubin et al., 2009; Setbon and Raude, 2010; Goodwin et al., 2011). Based on these findings and in line with the role of emotions in the *risk as feelings framework*, we expect that higher negative emotional reactions (i.e., worry) will be associated with higher perceived threat (H_2_). We also hypothesize that higher negative emotional reactions will be associated with higher engagement in self-protective behaviors (H_3_).

### Type of Threat

Emotional reactions and threat perception can be amplified or attenuated by specific characteristics of the hazard itself and how it is communicated. Characteristics such as perceived dreadfulness, controllability, and familiarity are among the most relevant ones (Fischhoff et al., 1978). Moreover, the coverage and the framing of the hazard in the media can influence these characteristics by making the threat and specific facets of it more salient and available in people’s minds (Tversky and Kahneman, 1973). For example, during the initial stage of the pandemic, the media often compared the coronavirus to the seasonal flu virus. However, while the two viruses share a similar symptomatology and behavioral interventions to reduce their spread (e.g., isolation, washing hands, distancing), they differ in other regards both from the medical and the psychological perception of the disease (Cowling et al., 2020; Haas, 2020). Medically, the lack of immunity and higher death rates in some subpopulations makes the coronavirus potentially more dangerous than the seasonal flu. Psychologically, at least at the beginning of the pandemic, the seasonal flu represented a more familiar and less dreadful hazard than the coronavirus.

Research showed that higher familiarity may produce an undervaluation of the risk because of the normalization of its presence in people’s life. Similarly, higher dread might cause an overvaluation of a threat by eliciting instinctive and negative emotional reactions (Slovic, 2000). Research on the availability heuristic suggests that heavy media coverage of a particular threat, such as the one related to the coronavirus, can make people overestimate the probability of death and increase the perception of risk of that specific hazard (Tversky and Kahneman, 1973; Lichtenstein et al., 1978; Cowling et al., 2020).

In sum, we expect to find that participants in our study will perceive higher worry (H_4_), higher perceived threat (H_5_), and will report higher engagement in self-protective behaviors (H_6_) when faced with information about the coronavirus (vs. seasonal flu). Moreover, we expect that the effect of the type of viral disease on engagement in self-protective behaviors will be serially mediated by both worry and perceived threat in line with the *risk as feelings* framework (H_7_).

### Type of Numerical Frame

By selecting and promoting (i.e., framing) some information rather than all information, the media can make some aspects of a story more or less salient and, in turn, bias people’s assessment of the threat (Entman, 1993). During the first stages of the coronavirus outbreak, for example, the media focused more on the information regarding the number of deaths (negative or loss frame) than the numbers of those who recovered (positive or gain frame; Hameleers, 2020).

Research in the health domain suggested that gain and loss framing can differently influence people’s decisions and behaviors, with gain frames being more effective in the context of preventive behaviors and loss frames being more effective in the context of health-promoting (e.g., screening) actions (Rothman and Salovey, 1997). However, reviews on different types of health behaviors are inconsistent in their findings and report little or contradictory effects of the two types of framing (O’Keefe and Jensen, 2007, 2009; Akl et al., 2011; Gallagher and Updegraff, 2012; O’Keefe and Nan, 2012). These inconsistencies extend also to research on the actual pandemic in which negative framing was found to be more effective in promoting action (Van Bavel et al., 2020), while other studies reported the opposite effect finding positive framing to be associated with higher support for strict preventive measures such as the lockdown (Hameleers, 2020).

However, in judgment and decision-making literature, evidence has been found regarding the ability of gain and loss frames to affect people’s emotional reactions. Gain frames generally elicit more positive emotional reactions, while loss frames elicit negative ones (Druckman and McDermott, 2008; Nabi et al., 2020). A recent meta-analysis (Nabi et al., 2020) highlighted also that emotional reactions mediate the relationship between the framing of a message and behavioral effects. This interpretation is in line with studies on the coronavirus pandemic investigating emotional reactions to positive and negative frames, including specific emotions such as frustration, fear, and powerlessness (Hameleers, 2020).

In the present study, we expect that providing negative numerical information (i.e., dead) vs. positive information (i.e., recovered) will lead participants to report higher levels of worry (H_8_), perceived threat (H_9_), and engagement in self-protective behaviors (H_10_). We also hypothesize, in line with the *risk as feelings* framework, that worry, and perceived threat will mediate the effect of the frame on the engagement in self-protective behaviors (H_11_).

Lastly, we also assessed several trait individual differences (subjective knowledge, trait emotional intelligence, conspiracy beliefs, trust in politics, media, and science) that have previously been linked to preventive actions in health-related decisions and studies on previous pandemics. Those individual differences were included with an exploratory purpose and are presented in the Supplementary Materials (see Sections 1, 3).

## Materials and Methods

### Participants

A total of 731 undergraduate students from Italy, Austria, and the United Kingdom participated in the study. Participants were excluded from the analysis if they (1) took either more or less than three standard deviations from the average time to complete the survey (*N* = 12), (2) did not fully complete the study (*N* = 110), or (3) failed the manipulation check (*N* = 62). Hence, the total sample comprises 547 participants (Table 1). Participants were recruited from a subject pool at the University of Trento (Italy) and the Behavioral Lab at Queen Mary University of London (United Kingdom), while the Austrian sample was recruited as part of a large undergraduate lecture at the University of Klagenfurt (Austria). They all received credits for their participation in the study. Ethical principles were respected following the Declaration of Helsinki and all participants provided their informed consent.

**TABLE 1:** Sample composition by Country.

	Italy	Austria	United Kingdom
Female	58.4%	81.5%	68.8%
Mean age	*M*_age_ = 25.9 years, *SD* = 8.47	*M*_age_ = 25.5 years, *SD* = 8.40	*M*_age_ = 23.4 years, *SD* = 5.20
Condition A	44 (26.5%)	56 (32.4%)	53 (25.5%)
Condition B	46 (27.7%)	48 (27.7%)	68 (32.7%)
Condition C	39 (23.5%)	38 (22%)	41 (19.7%)
Condition D	37 (22.3%)	31 (17.9%)	46 (22.1%)

*Italy N = 166, Austria N = 173, United Kingdom N = 208. Condition A: coronavirus-positive frame; Condition B: coronavirus-negative frame; Condition C: seasonal flu-positive frame; Condition D: seasonal flu-negative frame.*

### Design and Procedure

Data collection took place online from 11th to 18th of April, 2020. At that time, the three countries were all in a nationwide lockdown, even though it was implemented at different times and the rate of infections and mortality varied across the countries.

Participants in the three countries received an invitation via email to partake in a study about risk perception of diseases and public policies and were randomly assigned to one of the experimental conditions, resulting in a 2 (Viral Disease: coronavirus vs. seasonal flu) × 2 (Frame: positive vs. negative) × 3 (Country) between-subject design.

After reading the informed consent form and agreeing to take part, participants read a short text created to simulate the information provided by the media regarding one of the two viral diseases (coronavirus or seasonal flu). The term “coronavirus” was used instead of “COVID-19,” as it was prevalently used in the media at that time. In the positive frame condition, the number of people recovered from the viral disease was presented alongside the total number of people infected between October and March. The number of dead was used in the negative frame condition.

Participants’ emotional reactions and perceived threat of the viral disease, the public policies implemented by their national government, and the way the national media communicates about the disease were assessed. Perceived usefulness and dangerousness of the public policies and media communication were also assessed. Moreover, participants were asked how often they engage in self-protective behaviors (e.g., washing hands, coughing and sneezing in a tissue or flexed elbow). Participants in the coronavirus condition received information and answered questions referring only to COVID-19, while in the seasonal flu condition they received information and answered questions referring only to the seasonal flu. A manipulation check was also introduced before the demographic questions to confirm that participants had paid attention during the survey. The survey took around 20 min to complete.

The study design, manipulations, sample size, emotional reactions, and threat perception as main dependent variables were pre-registered on AsPredicted^1^. The texts for each condition and the items in English, German, and Italian are in the Supplementary Materials (see Section 2 and Supplementary Tables S1,S2). The datasets for the three countries are available on the OSF platform and are accessible through the following link: https://osf.io/uwv6r/?view_only=855c79250de8442b964f1bbd2f41626b.

### Materials

#### Emotional Reactions

Participants’ emotional reactions were assessed by asking how much they felt worried about the (1) viral disease, (2) public policies, and (3) media communication on a scale from 0 (Not worried) to 10 (Very worried). A new variable called “Worry” was created by combining the three items (Cronbach’s α_UK,AT,IT_ > 0.765).

#### Perceived Threat

To investigate participants’ perceived threat of the viral disease, the subjectively perceived likelihood of infection and perceived severity of the disease were assessed on a scale from 1 (Extremely low/Not dangerous at all) to 7 (Extremely high/Very dangerous). In line with studies on previous pandemics and Protection Motivation Theory, we created a variable called “Perceived threat” by multiplying the perceived severity of the disease by the subjectively perceived likelihood of infection (de Zwart et al., 2009; Leppin and Aro, 2009; Chang et al., 2016). To normalize the distribution of the new variable we performed a square root transformation. Thus, the new variable “Perceived threat” resulted in a scale from 1 (Low) to 7 (High). Perceived dangerousness and perceived usefulness of the public policies and the media communication were also assessed (1) in general, (2) for the national economy, (3) for the national social-emotional climate, and (4) for individuals’ physical health using a scale from 1 (Not dangerous/useful at all) to 7 (Very dangerous/useful). For each variable, one scale that included the four relevant items was created (Danger public policies: Cronbach’s α_UK,AT,IT_ > 0.791; Danger media communication: Cronbach’s α_UK,AT,IT_ > 0.886; Usefulness public policies: Cronbach’s α_UK,AT,IT_ > 0.697; Usefulness media communication: Cronbach’s α_UK,AT,IT_ > 0.840).

#### Behavior

Participants were asked to state how often they engage in protective behaviors from 1 (Never) to 7 (Always). Furthermore, participants’ perceived capability and control over the self-protective behaviors was assessed adapting two items from the Theory of Planned Behavior-TPB Questionnaire from Ajzen (2006).

#### Manipulation Check

To ensure participants paid attention while completing the survey, they were asked to indicate between four options (“Coronavirus”; “Seasonal flu”; “Measles”; “None of the options”) which viral disease they were asked to give their opinion about.

## Results

To test the effect of the manipulations (i.e., Viral Disease, Frame, and Country) on the three main variables (i.e., Behavior, Worry, and Perceived threat) we conducted a MANOVA. Subsequently, we ran a linear regression to test the predictors of Perceived threat for each country. Finally, to investigate our hypotheses concerning the relationship between our main dependent variables and the effect of the manipulations, we conducted a path analysis both for the total sample and for each country individually. *Post-hoc* power analyses indicated that we reached a power of at least 0.992 for all our tests.

### Effect of Frame, Viral Disease, and Country

A 2 (Viral Disease: coronavirus vs. seasonal flu) × 2 (Frame: positive vs. negative) × 3 (Country) MANOVA (Table 2) showed that Behavior, Worry, and Perceived threat varied significantly depending on Viral Disease, Frame, and Country.

**TABLE 2:** MANOVA of the effect of the manipulations on the three main dependent variables.

Source	Dependent variables	*df*	F	*p*	η*_*p*_*^2^
Viral disease	Behavior	1	18.74	<0.001	0.034
	Worry	1	387.32	<0.001	0.421
	Perceived threat	1	115.54	<0.001	0.178
	Wilks’ Lambda = 0.57, *F*(3, 532) = 132.45, *p* < 0.001				
Frame	Behavior	1	0.41	0.521	0.001
	Worry	1	8.48	0.004	0.016
	Perceived threat	1	2.01	0.157	0.004
	Wilks’ Lambda = 0.98, *F*(3, 532) = 2.89, *p* = 0.035				
Country	Behavior	2	1.40	0.247	0.008
	Worry	2	23.93	<0.001	0.117
	Perceived threat	2	11.64	<0.001	0.042
	Wilks’ Lambda = 0.87, *F*(6, 1064) = 12.81, *p* < 0.001				
Viral disease × Country	Behavior	2	1.55	0.213	0.006
	Worry	2	5.36	0.005	0.020
	Perceived threat	2	1.92	0.147	0.007
	Wilks’ Lambda = 0.97, *F*(6, 1064) = 2.68, *p* = 0.014				
Viral disease × Frame	Behavior	1	2.24	0.140	0.004
	Worry	1	0.25	0.616	0.001
	Perceived threat	1	0.08	0.783	<0.001
	Wilks’ Lambda = 0.99, *F*(3, 532) = 0.80, *p* = 0.512				
Frame × Country	Behavior	2	0.02	0.977	<0.001
	Worry	2	1.00	0.368	0.004
	Perceived threat	2	1.52	0.220	0.006
	Wilks’ Lambda = 0.99, *F*(6, 1064) = 0.85, *p* = 0.533				
Viral disease × Frame × Country	Behavior	2	1.73	0.178	0.006
	Worry	2	0.54	0.582	0.002
	Perceived threat	2	0.38	0.686	0.001
	Wilks’ Lambda = 0.99, *F*(6, 1064) = 1.00, *p* = 0.422				

*Viral Disease and Frame were coded orthogonally (Viral Disease: 0.5 = coronavirus, −0.5 = seasonal flu; Frame: 0.5 = positive frame, −0.5 = negative frame).*

#### Viral Disease

Participants in the coronavirus (vs. seasonal flu) condition indicated higher Perceived threat, Worry, and Behavior. These findings confirm part of our initial hypotheses (H_4_, H_5_, H_6_, H_8_) while others were rejected (H_9_, H_10_; see Table 6 for a summary of the hypotheses).

#### Framing

Results illustrate that participants were significantly more worried in the negative (vs. positive) frame condition. However, the type of frame did not affect participants’ Perceived threat or Behavior.

#### Country

Participants reported significantly higher Worry and Perceived threat in the United Kingdom sample compared to the Italian and Austrian sample (see Supplementary Table S4 for the main effect of “Country” on the complete list of our dependent variables).

Lastly, the MANOVA revealed a two-way interaction effect (Country × Viral Disease) on Worry (see Supplementary Table S5 for means and standard deviations). We performed a follow-up ANOVA to test the significance of the single comparisons. A Scheffè *post-hoc* test (De Mendiburu, 2020) showed that Italy reported significantly higher Worry than Austria in the coronavirus condition, but the two countries did not differ in the seasonal flu condition. The United Kingdom consistently reported the highest Worry in both Viral Disease conditions (see Table 3 and Supplementary Table S6 for significance and mean differences).

**TABLE 3:** Analysis of the interaction of Country and Viral Disease on Worry.

United Kingdom		Austria		Italy		*F*	*p*
		
Coronavirus	Seasonal flu	Coronavirus	Seasonal flu	Coronavirus	Seasonal flu		
5.27_a_	2.82_c_	3.87_b_	2.11_cd_	4.39_b_	1.68_d_	5.36	0.005

*Means with different subscripts differ at the p = 0.05 level by Scheffè test.*

### Predictors of Perceived Threat for Each Country

We performed a linear regression (Table 4) to assess, for each Country, the role of Worry, Perceived Dangerousness, and Usefulness of public policies and media communication as predictors of Perceived threat. Consistent with the literature that demonstrates a strong link between perceived risk and emotions, our results illustrated that Worry was the strongest predictor of participants’ Perceived threat in all countries. However, although the samples in the United Kingdom and Austria show similar results, in the Italian sample higher Perceived Usefulness of the public policies and the media communication, and higher Perceived Dangerousness of the media communication also predicted higher Perceived threat.

**TABLE 4:** Regression analysis for perceived threat.

Country		*B*	*SE*	*t*	*p*
United Kingdom	Worry	0.40	0.05	8.22	0.000
	Danger public policies	0.12	0.06	1.97	0.050
	Danger media communication	0.01	0.05	0.22	0.825
	Usefulness public policies	0.05	0.07	0.81	0.417
	Usefulness media communication	–0.04	0.05	–0.69	0.491
Austria	Worry	0.19	0.05	3.47	0.001
	Danger public policies	0.10	0.09	1.20	0.233
	Danger media communication	0.09	0.07	1.37	0.174
	Usefulness public policies	0.04	0.07	0.59	0.554
	Usefulness media communication	0.10	0.07	1.53	0.128
Italy	Worry	0.31	0.06	5.50	0.000
	Danger public policies	–0.04	0.08	–0.52	0.605
	Danger media communication	0.15	0.07	2.20	0.029
	Usefulness public policies	0.18	0.07	2.44	0.016
	Usefulness media communication	0.13	0.05	2.41	0.017

### Engagement in Self-Protective Behaviors: Direct and Indirect Effects

To test our hypotheses on the effect of the manipulations (i.e., Viral Disease and Frame) on self-protective behaviors, with Worry and Perceived threat as serial mediators, we used the entire sample for the analysis. Also, fitting our main model (Path model 2) separately for each country revealed a similar pattern of results (see Supplementary Materials Section 3 for details and other exploratory tested path models).

Although mean level differences exist between countries for some of the included variables, the regression analyses presented above have shown that Worry is a central predictor for Perceived threat for all countries.

Bivariate correlations between the variables of interest are presented in Table 5.

**TABLE 5:** Correlations among Perceived threat, Worry, and Behavior.

	Perceived threat	Worry	Behavior
Perceived threat	–		
Worry	0.586**	–	
Behavior	0.162**	0.198**	–

**p < 0.01.

**TABLE 6:** Summary of research hypotheses and results.

	Hypotheses	Results
H_1_	Higher perceived threat will be associated to higher engagement in self-protective behaviors	Supported
H_2_	Higher worry will be associated with higher perceived threat	Supported
H_3_	Higher worry will be associated with higher engagement in self-protective behaviors	Supported
H_4_	In the Coronavirus condition (vs. Seasonal Flu) participants will perceive higher worry	Supported
H_5_	In the Coronavirus condition (vs. Seasonal Flu) participants will report higher perceived threat	Not fully supported
H_6_	In the Coronavirus condition (vs. Seasonal Flu) participants will report higher engagement in self-protective behaviors	Supported
H_7_	The effect of Viral Disease manipulation on engagement in self-protective behaviors will be serially mediated by worry and perceived threat	Supported
H_8_	In the negative frame condition (vs. positive frame) participants will perceive higher worry	Supported
H_9_	In the negative frame condition (vs. positive frame) participants will report higher perceived threat	Not supported
H_10_	In the negative frame condition (vs. positive frame) participants will report higher engagement in self-protective behaviors	Not supported
H_11_	The effect of Frame manipulation on engagement in self-protective behaviors will be serially mediated by worry and perceived threat	Not fully supported

The results showed that Perceived threat was associated with higher engagement in self-protective behaviors (H_1_) and that higher Worry was associated with higher Perceived threat (H_2_). Moreover, higher emotional reactions were associated with higher engagement in self-protective behaviors (H_3_).

To investigate our hypotheses on direct and indirect effects of the manipulations we used Stata 14 (StataCorp, 2015) to conduct a path analysis using structure equation modeling (SEM). We first examined Path Model 1 to test H_7_ and H_11_. Specifically, we investigated the two indirect effects of our exogenous variables (i.e., Viral Disease and Frame) on the outcome variable (i.e., Behavior), serially mediated by Worry and Perceived threat, alongside with the direct effects of the exogenous variables on the outcome variable and the mediators. The resulting model was not significantly worse than the fully specified model, χ^2^ (1, *N* = 547) = 2.96, *p* = 0.085, and showed moderately good fit indices (RMSEA = 0.060, *p* = 0.292, CFI = 0.996, BIC = 6,634.0) according to Kline (2011). The results of the first model indicated that Viral Disease had a significant direct effect on Worry, *z* = 18.64, *p* < 0.001, 95% CI [2.07, 2.56], and Behavior, *z* = 3.03, *p* = 0.002, 95% CI [0.10, 0.45], but only marginally on Perceived threat, *z* = 1.95, *p* = 0.051, 95% CI [−0.01, 0.43]. Frame had a significant effect only on Worry, *z* = −3.29, *p* = 0.001, 95% CI [−0.64, −0.16]. These results support H_8_ but not H_9_ and H_10_.

**FIGURE 1:**
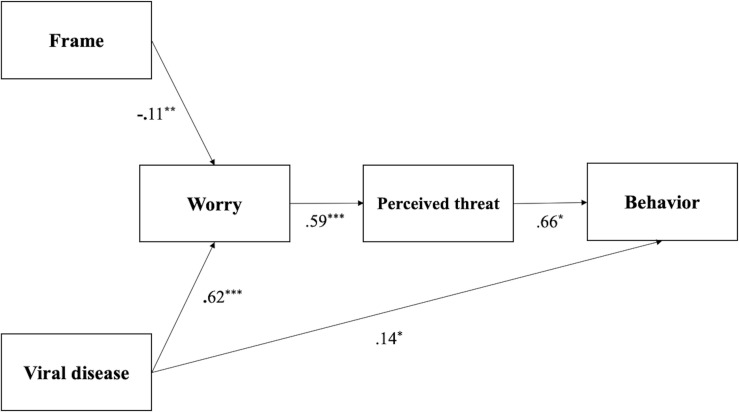
Path model testing the indirect effect of Frame and Viral Disease on Behavior and the direct effect of Viral Disease on Behavior. Coefficients presented are standardized. ^∗^*p* < 0.05, ^∗∗^*p* < 0.01, ^∗∗∗^*p* < 0.001.

We then removed the paths that did not show a significant effect to create a second, more parsimonious model (Figure 1). The second model tested the indirect effect of Frame and Viral Disease on the outcome variable (i.e., Behavior) and the direct effect of Viral Disease on Behavior (i.e., Path Model 2). The model showed a good fit, χ^2^ (4, *N* = 547) = 6.87, *p* = 0.143, RMSEA = 0.036, *p* = 0.632, the CFI = 0.995, BIC= 6,619.0, and was not significantly worse than Path Model 1, Δχ^2^ (3) = 3.91, *p* = 0.271.

Consistent with the hypothesis (H_7_), a positive and significant indirect effect emerged for Viral Disease on the engagement in self-protective behaviors serially mediated by Worry and Perceived threat, *z* = 2.22, *p* = 0.026, 95% CI [0.01, 0.14]. Being in the coronavirus (vs. seasonal flu) condition made participants more worried, which was related to a higher Perceived threat. Higher Perceived threat significantly and directly predicted higher self-reported engagement in self-protective behaviors. Our results also showed a significant direct effect of Viral Disease on the engagement in self-protective behaviors. The indirect effect of Frame on self-protective behavior with Worry and Perceived threat as serial mediators was only marginally significant, not supporting H_11,_ *z* = −1.85, *p* = 0.064, 95% CI [−0.03, 0.001]. However, being in the negative frame condition made participants experience more Worry and this was positively associated with higher Perceived threat, *z* = −3.19, *p* = 0.001, 95% CI [−1.83, −0.44].

## Discussion

In this study, we investigated different determinants of engagement in self-protective behaviors during the early stages of the COVID-19 pandemic in three European countries (Italy, Austria, and the United Kingdom). An overview of the hypotheses and results can be found in Table 6.

Perceived threat and negative emotional reaction (i.e., worry) have been identified as central predictors of self-reported preventive behaviors. Higher levels of perceived threat and higher worry were found to be associated with higher engagement in self-protective behaviors in all the countries sampled, and higher worry was consistently associated with higher perceived threat. Our results are consistent with psychological literature and studies on previous and the actual pandemic, indicating the perception of a threat as a prevailing factor in determining intention and effective implementation of protective behaviors (Brug et al., 2004; Rubin et al., 2009; Setbon and Raude, 2010; Goodwin et al., 2011; Sheeran et al., 2014; Niepel et al., 2020).

The role of worry is consistent with the “*risk as feelings*” framework in which affective reactions are considered to guide the judgment of risks and benefits (Slovic et al., 2002). Our results support also the argument that negative emotional reactions can have a positive effect on self-protective behaviors by their influence on risk perception. Communicators should be aware that conveying some level of worry in the population can be useful to enhance compliance with government interventions. We can speculate that a campaign aiming at underestimating the threat of the coronavirus, like the one implemented in the first stages of the pandemic by the British authorities (Conn et al., 2020) or as done by the American (Barth, 2020) and Brazilian (Kemeny, 2020) authorities, may lead citizens to not worry enough about the threat and consequently not protect against it sufficiently. On the other hand, it is possible that other emotions, such as fear or anxiety, can cause panic, and lead to overreactions, such as exaggerated protective behaviors, discrimination toward groups associated with the threat and, mental illness symptoms (Yang and Cho, 2017; Taylor, 2019; Depoux et al., 2020). Thus, media communication and policies should be careful in tailoring messages for the population that induces a commensurate emotional reaction and risk perception.

Our experiment aimed also at understanding if the way the media addressed the pandemic might have affected threat perception, emotional reaction, and compliance with the behavioral indications propagated by the WHO. Information about the coronavirus or the seasonal flu (Viral Disease manipulation) reporting the number of those who died (Negative Frame) or those who recovered (Positive Frame) was presented to participants to mimic actual media communication at the time of the study.

Results showed a significant indirect effect of the Viral Disease manipulation on behavior serially mediated by worry and perceived threat. People in the coronavirus condition were more worried, which was related to a higher perceived threat and, subsequently, higher compliance with self-protective behaviors. These results are in line with the availability heuristic and the risk profile of the two diseases (Tversky and Kahneman, 1973; Fischhoff et al., 1978; Cowling et al., 2020). Higher dreadfulness and heavy media coverage of a particular threat, such as the one related to the coronavirus, can make people overestimate the probability of death and increase the perception of risk. Conversely, higher familiarity with a threat (e.g., seasonal flu), and lower media attention may produce an undervaluation of the risk (Tversky and Kahneman, 1973; Fischhoff et al., 1978; Lichtenstein et al., 1978; Slovic, 2000; Cowling et al., 2020). It is therefore advisable to stay away from a comparison that can trigger people’s use of heuristics judgment and lead to an underestimation of the risk.

Our results also showed that people in the Negative Frame condition (vs. Positive Frame) reported higher levels of worry, consistent with previous research (Peters et al., 2006a; Druckman and McDermott, 2008; Hameleers, 2020). Higher worry, in turn, was associated with a higher perceived threat, which is in line with previous studies showing the effect of the frame on other kinds of emotional reactions in health-related behaviors (Peters et al., 2006a). Finally, although previous literature shows an effect of framing on preventive actions in the COVID-19 pandemic (Hameleers, 2020; Van Bavel et al., 2020), our results did not show a significant impact of framing on self-protective behaviors. However, previous research on the coronavirus pandemic tested mainly equivalency frames (Hameleers, 2020). An equivalency frame consists of offering the same information with different presentation and organization formats following the example of the studies on framing from Tversky and Kahneman (1981). In their study, participants read one of two numerically identical scenarios regarding possible programs aiming to combat an Asian disease, presenting either the number of people who could die or the number of who could be saved. The different framing elicited a preference reversal and a different attitude toward risk.

In our paper, we choose instead to test a different type of framing by reporting the real numbers of deaths and recovered, therefore using a frame that is best identified in the group of “emphasis frames” (Entman, 1993). Emphasis frames do not present equivalent numerical information but focus on a different facet of events making some information more salient than others. We believe that this type of framing allowed us to better mimic how the media report the numbers of dead and recovered in the early stage of the pandemic. This conceptual difference could partially account for our results. Indeed, using actual numbers can provide a more realistic approach but is subject to interference by previous knowledge of the number of infections, deaths, and recovered by the participants.

### Limitation and Future Directions

In our study, we considered a comprehensive affective reaction to the pandemic including not only the reactions to the disease but also to the public policies and the media communication. Focusing on such a general emotional reaction may allow inclusive inferences but also lacks specificity. In addition, we focused solely on worry as a negative emotional reaction as it was identified as main driver of threat perception in prior studies (Peters et al., 2006b). In future research, the emotional reactions to the pandemic can be assessed both in a general and more specific way and other emotions (e.g., fear, frustration, powerlessness) should be taken into consideration.

We calculated the perceived threat multiplying the perceived likelihood of contagion and the perceived disease severity following works on previous pandemics and the PTM model. However, different approaches to assess risk perception, as the Tripartite Model of Risk Perception (TRIRISK; Ferrer et al., 2016), can be tested in future studies. Furthermore, we assessed the perceived severity of the disease in a generic manner (i.e., “how dangerous is the coronavirus”) while the likelihood of contagion was directly addressed to the participant (i.e., “What is the probability that you will get infected by the coronavirus in the next month?”). The generic format of the severity question gave participants greater freedom of interpretation but makes it impossible to know whether participants were referring to themselves or to others. However, perceived severity correlated positively with the engagement in self-protective behavior, which was addressed directly to the participant. This gives us reasons to think that, overall, participants interpreted the severity question to include personal danger to themselves.

The framing manipulation was presented only at the beginning of the survey. In future studies, the manipulation should be presented more than once, or recalled in crucial questions, to better recall the frame. The actual numbers shared by the primary national media were used in our manipulation. Although these numbers might be slightly different than the factual number of deaths or recovered because of the difficulties in assessing them, we decided to report those numbers to have a more ecological representation of reality.

Finally, future research should replicate these results with larger and more representative samples from the general population.

## Data Availability Statement

The datasets presented in this study can be found in online repositories. The names of the repository/repositories and accession number(s) can be found below: https://osf.io/uwv6r/?view_only=855c79250de8442b964f1bbd2f41626b and doi: 10.17605/OSF.IO/UWV6R.

## Ethics Statement

Ethical review and approval was not required for the study on human participants in accordance with the local legislation and institutional requirements. The patients/participants provided their written informed consent to participate in this study.

## Author Contributions

GP and MV ran the data collection, organized the database, and wrote the first draft of the manuscript. MV performed the statistical analysis. All authors contributed to the conception and design of the study, data interpretation, manuscript revision, and also read and approved the submitted version.

## Conflict of Interest

The authors declare that the research was conducted in the absence of any commercial or financial relationships that could be construed as a potential conflict of interest.

## Footnotes

1 https://aspredicted.org/76tj5.pdf
