# Supplementary material for: Worry, Perceived Threat and Media Communication as Predictors of Self-Protective Behaviors During the COVID-19 Outbreak in Europe
Source: Front Psychol. 2021 Feb 16;12:577992. doi: 10.3389/fpsyg.2021.577992 (PMC7921485; doi:10.3389/fpsyg.2021.577992)
Supplement: Supplementary file 1 [file Data_Sheet_1.docx]

**Supplementary materials**

**Section 1. Trait individual differences**

- 1. ***Literature background.***

Apart from investigating the effects of framing and the type of disease, our study was designed with specific trait individual differences in mind which have previously been linked to preventive actions in health-related decisions and studies on previous pandemics. The motivation for including these individual differences also stems from the observation that individuals differ in their acceptance of self-protective behavior.

Considering the central role of feelings and affect in risk perception and health-related behaviors, we wanted to investigate the role of the ability to self-regulate one’s emotional reactions. Trait emotional intelligence (trait EI) is defined as the ability to identify, manage, and use emotions (Petrides et al., 2007). Individuals with high trait EI tend to regulate emotions more adaptively, are more able to deal with and downregulate negative affect under stress, are less influenced by irrelevant stimuli and perceive events as less threatening (Peña et al., 2015; Rubaltelli et al., 2020; Scrimin & Rubaltelli, 2019; Sevdalis et al., 2007). Based on that, we expect that participants with high trait EI will show a weaker emotional reaction and subsequently lower risk perception. Moreover, some dimensions of the construct have also been related to preventive (e.g., wellness maintenance) behaviors (Fernández-Abascal & Martín-Díaz, 2015; Mikolajczak et al., 2015), but to the best of our knowledge no study investigated the relationship with self-protective behaviors specifically. Nonetheless, we expect that higher trait EI will be associated with higher engagement in self-protective behaviors.

Moreover, perceived behavioral control over the action can also play a significant role in behavior engagement. According to Ajzen’s Theory of Planned Behaviors (Ajzen, 1991), people need to feel able to control and have the resources to master the activity to carry out a behavior. We predict that those who feel to have high control over their engagement in self-protective behaviors will report higher effective compliance with those. Indeed, correlations between perceived behavioral control and health-related behaviors have been found, although those correlations were low (Ajzen, 2011; McEachan et al., 2011).

Additionally, we also examined the possible effects of personal values and worldviews such as conspiracy beliefs, trust in politics, media and science, and knowledge about the disease and the non-pharmaceutical measures taken by the authorities. Since the beginning of the pandemic, high levels of fake news and conspiracy-frame narratives around the origins of the virus as well as remedies against it (e.g., the virus was created in a Chinese lab as a biological weapon; was caused by 5G electromagnetic waves; disinfectant injection can be used as a treatment) were disseminated among the media (Cuan-Baltazar et al., 2020; Depoux et al., 2020; Kouzy et al., 2020; Mian & Khan, 2020; Pennycook et al., 2020). The uncontrollable proliferation of misinformation and confusing news can make people adopt ineffective remedies and refrain from adhering to recommended self-protective behaviors (Brainard & Hunter, 2020; Pennycook et al., 2020; Taylor, 2019). Believing in conspiracy theories has been linked to negative health behavior such as a preference for alternative medicine and a lower likelihood to get influenza shots and vaccines against H1N1 (Lohiniva et al., 2014; Oliver & Wood, 2014; Setbon & Raude, 2010). In line with this, we expect to find lower engagement in self-protective behaviors in those more prone to endorse conspiracy theories.

This misinformation epidemic (Kouzy et al., 2020) and the different strategies implemented by governments can reduce public trust and bias citizens knowledge about the situation. However, both trust and knowledge play a key role in guiding citizens’ risk perception (Siegrist & Cvetkovich, 2000). Trust in authorities, in media and science has been found relevant in enhancing compliance with recommended behaviors and vaccinations during previous pandemics (Kim & Song, 2017; Plohl & Musil, 2020; Prati et al., 2011; Siegrist & Zingg, 2014; Yang & Cho, 2017). Higher engagement in behaviors and lower risk perception were also found in individuals with higher disease-related knowledge (Brug et al., 2004; Rolison & Hanoch, 2015; Yang & Cho, 2017).

***1.2 Materials***

Participants' subjectively perceived knowledge about 1) the viral disease, 2) the public policies, and 3) the self-protective behaviors was measured by asking participants to rate from 1 (Nothing at all) to 7 (Very much) how much they think they know about the three topics. The three items were combined in a single variable (Knowledge). Trait emotional intelligence was assessed with the TEIQue-SF (Petrides, 2009) with 30 items using a 7-point scale ranging from 1 (Completely Disagree) to 7 (Completely Agree). The 15-item Generic Conspiracist Beliefs Scale (GCB - Brotherton et al., 2013) was used to measure participants’ beliefs in conspiracies at a general level on a 5-point scale from 1 (Definitely not true) to 5 (Definitely true). Participants’ trust in politics (Trust in politics) was assessed by asking how much they trusted national (legal system, police, politician, political parties, the Department of Health) and international institutions (WHO and European Parliament) on a scale from 0 (Not trust at all) to 10 (Complete trust) adapted from Ervasti & Ervasti (2008). To measure trust in media (Trust in media), two sub-scales from the ADTRUST scale were adapted (Soh et al., 2009). The scales assessed participants’ perceived reliability and usefulness of the information conveyed in national media with 13 adjectives (e.g., Honest, Credible, Valuable, etc.) on a scale from 1 (Completely Disagree) to 7 (Completely Agree). Finally, a scale from McCright, Dentzman, Charters & Dietz (2013) was adapted to understand participants’ trust in science (Trust in science), measured by ratings from 1 (Completely distrust) to 5 (Completely trust) how much participants trusted scientists to create unbiased and useful knowledge with four items. Cronbach’s alphas were acceptable for all of the scales and created variables (Cronbach's _UK,AT,IT_ > .739).

***1.3 References***

Ajzen, I. (1991). The theory of planned behavior. *Organizational Behavior and Human Decision Processes*, *50*(2), 179–211. https://doi.org/10.1016/0749-5978(91)90020-T

Ajzen, I. (2011). The theory of planned behaviour: Reactions and reflections. *Psychology & Health*, *26*(9), 1113–1127. https://doi.org/10.1080/08870446.2011.613995

Brainard, J., & Hunter, P. R. (2020). Misinformation making a disease outbreak worse: Outcomes compared for influenza, monkeypox, and norovirus. *SIMULATION*, *96*(4), 365–374. https://doi.org/10.1177/0037549719885021

Brotherton, R., French, C. C., & Pickering, A. D. (2013). Measuring Belief in Conspiracy Theories: The Generic Conspiracist Beliefs Scale. *Frontiers in Psychology*, *4*. https://doi.org/10.3389/fpsyg.2013.00279

Brug, J., Aro, A. R., Oenema, A., de Zwart, O., Richardus, J. H., & Bishop, G. D. (2004). SARS Risk Perception, Knowledge, Precautions, and Information Sources, the Netherlands. *Emerging Infectious Diseases*, *10*(8), 1486–1489. https://doi.org/10.3201/eid1008.040283

Cuan-Baltazar, J. Y., Muñoz-Perez, M. J., Robledo-Vega, C., Pérez-Zepeda, M. F., & Soto-Vega, E. (2020). Misinformation of COVID-19 on the Internet: Infodemiology Study. *JMIR Public Health and Surveillance*, *6*(2), e18444. https://doi.org/10.2196/18444

Depoux, A., Martin, S., Karafillakis, E., Preet, R., Wilder-Smith, A., & Larson, H. (2020). The pandemic of social media panic travels faster than the COVID-19 outbreak. *Journal of Travel Medicine*, *27*(3). https://doi.org/10.1093/jtm/taaa031

Ervasti, H., & Ervasti, H. (2008). *Nordic Social Attitudes in a European Perspective*. Edward Elgar Publishing.

Fernández-Abascal, E. G., & Martín-Díaz, M. D. (2015). Dimensions of emotional intelligence related to physical and mental health and to health behaviors. *Frontiers in Psychology*, *6*. https://doi.org/10.3389/fpsyg.2015.00317

Kim, C. W., & Song, H. R. (2017). Structural Relationships among Public’s Risk Characteristics, Trust, Risk Perception and Preventive Behavioral Intention—The Case of MERS in Korea -. *Crisis and Emergency Management*, *13*(6), 85–95. https://doi.org/10.14251/crisisonomy.2017.13.6.85

Kouzy, R., Abi Jaoude, J., Kraitem, A., El Alam, M. B., Karam, B., Adib, E., Zarka, J., Traboulsi, C., Akl, E. W., & Baddour, K. (2020). Coronavirus Goes Viral: Quantifying the COVID-19 Misinformation Epidemic on Twitter. *Cureus*, *12*(3). https://doi.org/10.7759/cureus.7255

Lohiniva, A.-L., Barakat, A., Dueger, E., Restrepo, S., & Aouad, R. E. (2014). A Qualitative Study of Vaccine Acceptability and Decision Making among Pregnant Women in Morocco during the A (H1N1) pdm09 Pandemic. *PLOS ONE*, *9*(10), e96244. https://doi.org/10.1371/journal.pone.0096244

McCright, A. M., Dentzman, K., Charters, M., & Dietz, T. (2013). The influence of political ideology on trust in science. *Environmental Research Letters*, *8*(4), 044029. https://doi.org/10.1088/1748-9326/8/4/044029

McEachan, R. R. C., Conner, M., Taylor, N. J., & Lawton, R. J. (2011). Prospective prediction of health-related behaviours with the Theory of Planned Behaviour: A meta-analysis. *Health Psychology Review*, *5*(2), 97–144. <https://doi.org/10.1080/17437199.2010.521684>

Mian, A., & Khan, S. (2020). Coronavirus: The spread of misinformation. *BMC Medicine*, *18*(1), 89. https://doi.org/10.1186/s12916-020-01556-3

Mikolajczak, M., Avalosse, H., Vancorenland, S., Verniest, R., Callens, M., van Broeck, N., Fantini-Hauwel, C., & Mierop, A. (2015). A nationally representative study of emotional competence and health. *Emotion*, *15*(5), 653–667. https://doi.org/10.1037/emo0000034

Oliver, J. E., & Wood, T. (2014). Medical Conspiracy Theories and Health Behaviors in the United States. *JAMA Internal Medicine*, *174*(5), 817–818. https://doi.org/10.1001/jamainternmed.2014.190

Peña-Sarrionandia, A., Mikolajczak, M., & Gross, J. J. (2015). Integrating emotion regulation and emotional intelligence traditions: A meta-analysis. *Frontiers in Psychology*, *6*. https://doi.org/10.3389/fpsyg.2015.00160

Pennycook, G., McPhetres, J., Zhang, Y., Lu, J. G., & Rand, D. G. (2020). *Fighting COVID-19 misinformation on social media: Experimental evidence for a scalable accuracy nudge intervention* [Preprint]. PsyArXiv. https://doi.org/10.31234/osf.io/uhbk9

Petrides, K. V. (2009). Psychometric Properties of the Trait Emotional Intelligence Questionnaire (TEIQue). In J. D. A. Parker, D. H. Saklofske, & C. Stough (Eds.), *Assessing Emotional Intelligence: Theory, Research, and Applications* (pp. 85–101). Springer US. https://doi.org/10.1007/978-0-387-88370-0_5

Petrides, K. V., Pita, R., & Kokkinaki, F. (2007). The location of trait emotional intelligence in personality factor space. *British Journal of Psychology*, *98*(2), 273–289. https://doi.org/10.1348/000712606X120618

Plohl, N., & Musil, B. (2020). Modeling compliance with COVID-19 prevention guidelines: The critical role of trust in science. *Psychology, Health & Medicine*, *0*(0), 1–12. https://doi.org/10.1080/13548506.2020.1772988

Prati, G., Pietrantoni, L., & Zani, B. (2011). Compliance with recommendations for pandemic influenza H1N1 2009: The role of trust and personal beliefs. *Health Education Research*, *26*(5), 761–769. https://doi.org/10.1093/her/cyr035

Rolison, J. J., & Hanoch, Y. (2015). Knowledge and risk perceptions of the Ebola virus in the United States. *Preventive Medicine Reports*, *2*, 262–264. https://doi.org/10.1016/j.pmedr.2015.04.005

Rubaltelli, E., Priolo, G., Scrimin, S., & Moscardino, U. (2020). Media Exposure to Terrorism and Perception of Immigrants as a Threat: The Role of Emotional Intelligence and Psychophysiological Self-Regulation. *Risk Analysis*. https://doi.org/10.1111/risa.13498

Scrimin, S., & Rubaltelli, E. (2019). Dehumanization after terrorism: The role of psychophysiological emotion regulation and trait emotional intelligence. *Current Psychology*. https://doi.org/10.1007/s12144-019-00189-x

Setbon, M., & Raude, J. (2010). Factors in vaccination intention against the pandemic influenza A/H1N1. *European Journal of Public Health*, *20*(5), 490–494. https://doi.org/10.1093/eurpub/ckq054

Sevdalis, N., Petrides, K. V., & Harvey, N. (2007). Trait emotional intelligence and decision-related emotions. *Personality and Individual Differences*, *42*(7), 1347–1358. <https://doi.org/10.1016/j.paid.2006.10.012>

Siegrist, M., & Cvetkovich, G. (2000). Perception of Hazards: The Role of Social Trust and Knowledge. *Risk Analysis*, *20*(5), 713–720. https://doi.org/10.1111/0272-4332.205064

Siegrist, M., & Zingg, A. (2014). The Role of Public Trust During Pandemics: Implications for Crisis Communication. *European Psychologist*, *19*(1), 23–32. https://doi.org/10.1027/1016-9040/a000169

Soh, H., Reid, L. N., & King, K. W. (2009). Measuring Trust In Advertising. *Journal of Advertising*, *38*(2), 83–104. https://doi.org/10.2753/JOA0091-3367380206

Taylor, S. (2019). *The Psychology of Pandemics: Preparing for the Next Global Outbreak of Infectious Disease*. Cambridge Scholars Publishing.

Yang, S., & Cho, S.-I. (2017). Middle East respiratory syndrome risk perception among students at a university in South Korea, 2015. *American Journal of Infection Control*, *45*(6), e53–e60. <https://doi.org/10.1016/j.ajic.2017.02.013>

**Section 2. Original materials of the manipulations and items translated in English, German and Italian.**

***Table S1. Original materials of the Frame and Viral Disease manipulations in the three countries.***

| Country | Viral disease | Frame |  |
| --- | --- | --- | --- |
| UK | Coronavirus | Positive | “From October 2019 to March 2020, people around the world have been infected by the new coronavirus, and some of these people have recovered.   In the UK, this year it has been estimated 11,658 cases and 135 people have recovered. “ |
|  |  | Negative | “From October 2019 to March 2020, several people around the world have been infected by the new coronavirus, and some of these people have died.  In the UK, this year it has been estimated 11,658 cases and 578 people have died.” |
|  | Seasonal flu | Positive | “From October to March every year, as also in 2020, several people around the world are infected by the seasonal flu, and some of these people recovered.  In the UK, this year it has been estimated that 6,775,000 people have been infected and 6,758,220 have recovered.” |
|  |  | Negative | “From October to March every year, as also in 2020, several people around the world are infected by the seasonal flu, and some people die.  In the UK, this year it has been estimated that 6,775,000 people have been infected and 16,780 have died.” |
| Austria | Coronavirus | Positive | “Im Zeitraum von Oktober 2019 bis März 2020 haben sich Menschen weltweit mit dem neuen Coronavirus infiziert und einige dieser Menschen sind bereits wieder gesund.  In Österreich haben Schätzungen dieses Jahr ergeben, dass es bisher 7.196 Fälle gab und 225 Menschen wieder gesund wurden.” |
|  |  | Negative | “Im Zeitraum von Oktober 2019 bis März 2020 haben sich Menschen weltweit mit dem neuen Coronavirus infiziert und einige dieser Menschen sind bereits gestorben.  In Österreich haben Schätzungen dieses Jahr ergeben, dass es bisher 7.196 Fälle gab und 58 Menschen gestorben sind.” |
|  | Seasonal flu | Positive | “Jedes Jahr von Oktober bis März, so auch 2020, infizieren sich Menschen weltweit mit dem saisonalen Grippevirus und einige dieser Menschen werden wieder gesund.  In Österreich haben Schätzungen dieses Jahr ergeben, dass sich bisher 890.000 Menschen angesteckt haben und 887.888 Menschen wieder gesund worden sind.” |
|  |  | Negative | “Jedes Jahr von Oktober bis März, so auch 2020, infizieren sich Menschen weltweit mit dem saisonalen Grippevirus und einige dieser Menschen sterben.  In Österreich haben Schätzungen dieses Jahr ergeben, dass sich bisher 890.000 Menschen angesteckt haben und 2.112 Menschen gestorben sind.” |
| Italy | Coronavirus | Positive | “Da Ottobre 2019 a Marzo 2020, diverse persone nel mondo sono state contagiate dal nuovo coronavirus e alcune di queste persone sono guarite.  In Italia, ci sono stati 86.498 casi di cui 10.950 guariti.” |
|  |  | Negative | “Da Ottobre 2019 a Marzo 2020, diverse persone nel mondo sono state contagiate dal nuovo coronavirus e alcune di queste persone sono decedute.  In Italia, ci sono stati 86.498 casi di cui 9.134 deceduti.” |
|  | Seasonal flu | Positive | “Da Ottobre a Marzo ogni anno, come anche nel 2020, diverse persone nel mondo vengono contagiate dall'influenza stagionale e alcune di queste persone guariscono.  In Italia, si stima che quest'anno 6.027.500 persone siano state contagiate e che 6.019.500 siano guarite.” |
|  |  | Negative | “Da Ottobre a Marzo ogni anno, come anche nel 2020, diverse persone nel mondo vengono contagiate dall'influenza stagionale e alcune di queste persone muoiono.  In Italia, si stima che quest'anno 6.027.500 persone siano state contagiate e che 8.000 siano morte.” |

***Table S2. Complete list of items and measuring scales for the three countries.***

| Country | Variable | Level | Item | Responses scale |
| --- | --- | --- | --- | --- |
| UK | Emotional Reaction | disease | When I think about the coronavirus/seasonal flu I feel... | (0) Not Worried/(10) Very Worried |
|  |  | public policies | When I think about the public policies implemented by the UK Government to limit the spread of coronavirus/seasonal flu I feel... | (0) Not Worried/(10) Very Worried |
|  |  | media | When I think about what has been told about the coronavirus/seasonal flu in the UK media (journals, an online newspaper, television news) I feel... | (0) Not Worried/(10) Very Worried |
|  | Likelihood to get infected |  | What is the probability that you will get infected by coronavirus/seasonal flu in the next month? | (1) Extremely low/(7) Extremely high |
|  | Severity of the disease |  | How dangerous is the coronavirus/seasonal flu? | (1) Not dangerous at all/(7) Very dangerous |
|  | Behavior Capability |  | For me to follow the propagated behavior in the forthcoming month would be... | (1) Impossible/(7) Possible |
|  | Behavior Control |  | It is mostly up to me to follow the propagated behavior in the forthcoming month. | (1) Strongly disagree/(6) Strongly agree |
|  | Behavior |  | Please indicate how often you engage in the propagated behavior using a scale from 1 to 7 where 1 represents “Never” and 7 represents “Always”. | (1) Never/(7) Always |
|  | Danger Public Policies | in general | The UK Government has implemented public policies to limit the spread of the coronavirus/seasonal flu. In your opinion, how dangerous are these public policies...(1) in general? | (1) Not dangerous at all/(7) Extremely dangerous |
|  |  | for national economy | The UK Government has implemented public policies to limit the spread of the coronavirus/seasonal flu. In your opinion, how dangerous are these public policies...(2) for the national economy? | (1) Not dangerous at all/(7) Extremely dangerous |
|  |  | for national social-emotional climate | The UK Government has implemented public policies to limit the spread of the coronavirus/seasonal flu. In your opinion, how dangerous are these public policies...(3) for the national social-emotional climate? | (1) Not dangerous at all/(7) Extremely dangerous |
|  |  | for individual physical health | The UK Government has implemented public policies to limit the spread of the coronavirus/seasonal flu. In your opinion, how dangerous are these public policies...(4) for individuals’ physical health? | (1) Not dangerous at all/(7) Extremely dangerous |
|  | Danger Media Communication | in general | How much do you perceive the way the UK media talk about the coronavirus/seasonal flu as dangerous...(1) in general? | (1) Not dangerous at all/(7) Extremely dangerous |
|  |  | for national economy | How much do you perceive the way the UK media talk about the coronavirus/seasonal flu as dangerous...(2) for the national economy? | (1) Not dangerous at all/(7) Extremely dangerous |
|  |  | for national social-emotional climate | How much do you perceive the way the UK media talk about the coronavirus/seasonal flu as dangerous...(3) for the national social-emotional climate? | (1) Not dangerous at all/(7) Extremely dangerous |
|  |  | for individual physical health | How much do you perceive the way the UK media talk about the coronavirus/seasonal flu as dangerous...(4) for individuals’ physical health? | (1) Not dangerous at all/(7) Extremely dangerous |
|  | Usefulness Public Policies | in general | The UK Government has implemented public policies to limit the spread of the coronavirus/seasonal flu. In your opinion, how useful are these public policies...(1) in general? | (1) Not useful at all/(7) Extremely useful |
|  |  | for national economy | The UK Government has implemented public policies to limit the spread of the coronavirus/seasonal flu. In your opinion, how useful are these public policies...(2) for the national economy? | (1) Not useful at all/(7) Extremely useful |
|  |  | for national social-emotional climate | The UK Government has implemented public policies to limit the spread of the coronavirus/seasonal flu. In your opinion, how useful are these public policies...(3) for the national social-emotional climate? | (1) Not useful at all/(7) Extremely useful |
|  |  | for individual physical health | The UK Government has implemented public policies to limit the spread of the coronavirus/seasonal flu. In your opinion, how useful are these public policies...(4) for individuals’ physical health? | (1) Not useful at all/(7) Extremely useful |
|  | Usefulness Media Communication | in general | How much do you perceive the way the UK media talk about the coronavirus/seasonal flu as useful...(1) in general? | (1) Not useful at all/(7) Extremely useful |
|  |  | for national economy | How much do you perceive the way the UK media talk about the coronavirus/seasonal flu as useful...(2) for the national economy? | (1) Not useful at all/(7) Extremely useful |
|  |  | for national social-emotional climate | How much do you perceive the way the UK media talk about the coronavirus/seasonal flu as useful...(3) for the national social-emotional climate? | (1) Not useful at all/(7) Extremely useful |
|  |  | for individual physical health | How much do you perceive the way the UK media talk about the coronavirus/seasonal flu as useful...(4) for individuals’ physical health? | (1) Not useful at all/(7) Extremely useful |
|  | Knowledge | for public policies | How much do you think you know about the public policies implemented by the UK Government to limit the spread of the coronavirus/seasonal flu? (1) | (1) Nothing at all/(7) Very much |
|  |  | for behavior | How much do you think you know about the propagated behaviors to limit the spread of the coronavirus/seasonal flu? (2) | (1) Nothing at all/(7) Very much |
|  |  | in general | How much do you think you know about the coronavirus/seasonal flu? (3) | (1) Nothing at all/(7) Very much |
|  | Manipulation Check |  | Please indicate, in the list below, which viral disease you were asked to give your opinion about: | (1) Coronavirus/(2) Seasonal flu/(3) Measles/(4) None of the options |
| Austria | Emotional Reaction | disease | Wenn ich über das Coronavirus/Saisonalen Grippevirus nachdenke fühle ich (mich)... | (0) Überhaupt nicht besorgt/(10) Sehr besorgt |
|  |  | public policies | Wenn ich über die, von der österreichischen Regierung vorgenommenen, Maßnahmen gegen die weitere Ausbreitung des Coronavirus/Saisonalen Grippevirus nachdenke, dann fühle ich (mich)... | (0) Überhaupt nicht besorgt/(10) Sehr besorgt |
|  |  | media | Wenn ich darüber nachdenke, was in den österreichischen Medien (Zeitschriften, online Zeitungen, Fernsehen) über das Coronavirus/Saisonalen Grippevirus berichtet wird, dann fühle ich (mich)... | (0) Überhaupt nicht besorgt/(10) Sehr besorgt |
|  | Likelihood to get infected |  | Wie wahrscheinlich ist es, dass Sie sich im nächsten Monat mit dem Coronavirus/Saisonalen Grippevirus infizieren? | (1) Sehr niedrig/(7) Sehr hoch |
|  | Severity of the disease |  | Wie gefährlich ist das Coronavirus/Saisonalen Grippevirus? | (1) Überhaupt nicht gefährlich /(7) Sehr gefährlich |
|  | Behavior Capability |  | Die empfohlenen Verhaltensweisen im kommenden Monat einzuhalten, wäre für mich... | (1) Unmöglich/(7) Möglich |
|  | Behavior Control |  | Ob ich im kommenden Monat die empfohlenen Verhaltensweisen einhalten werde, habe ich größtenteils selbst in der Hand. | (1) Stimme gar nicht zu /(6) Stimme sehr zu |
|  | Behavior |  | Bitte geben Sie auf einer Skala von 1 bis 7 an, wie häufig sie die zuvor genannten Verhaltensweisen anwenden, wobei 1 für "Nie" und 7 für "Immer" steht | (1) Nie/(7) Immer |
|  | Danger Public Policies | in general | Die österreichische Regierung hat Maßnahmen vorgenommen, um die weitere Ausbreitung des Coronavirus/Saisonalen Grippevirus einzugrenzen. Wie gefährlich sind diese staatlichen Maßnahmen Ihrer Meinung nach...(1) im Allgemeinen? | (1) Überhaupt nicht gefährlich/(7) Sehr gefährlich |
|  |  | for national economy | Die österreichische Regierung hat Maßnahmen vorgenommen, um die weitere Ausbreitung des Coronavirus/Saisonalen Grippevirus einzugrenzen. Wie gefährlich sind diese staatlichen Maßnahmen Ihrer Meinung nach...(2) für die nationale Wirtschaft? | (1) Überhaupt nicht gefährlich/(7) Sehr gefährlich |
|  |  | for national social-emotional climate | Die österreichische Regierung hat Maßnahmen vorgenommen, um die weitere Ausbreitung des Coronavirus/Saisonalen Grippevirus einzugrenzen. Wie gefährlich sind diese staatlichen Maßnahmen Ihrer Meinung nach…(3) für das nationale sozial-emotionale Klima? | (1) Überhaupt nicht gefährlich/(7) Sehr gefährlich |
|  |  | for individual physical health | Die österreichische Regierung hat Maßnahmen vorgenommen, um die weitere Ausbreitung des Coronavirus/Saisonalen Grippevirus einzugrenzen. Wie gefährlich sind diese staatlichen Maßnahmen Ihrer Meinung nach...(4) für die physische Gesundheit des Einzelnen? | (1) Überhaupt nicht gefährlich/(7) Sehr gefährlich |
|  | Danger Media Communication | in general | Für wie gefährlich halten Sie die Art und Weise wie die österreichischen Medien über das Coronavirus/Saisonalen Grippevirus berichten...(1) im Allgemeinen? | (1) Überhaupt nicht gefährlich/(7) Sehr gefährlich |
|  |  | for national economy | Für wie gefährlich halten Sie die Art und Weise wie die österreichischen Medien über das Coronavirus/Saisonalen Grippevirus berichten... (2) für die nationale Wirtschaft? | (1) Überhaupt nicht gefährlich/(7) Sehr gefährlich |
|  |  | for national social-emotional climate | Für wie gefährlich halten Sie die Art und Weise wie die österreichischen Medien über das Coronavirus/Saisonalen Grippevirus berichten... (3) für das nationale sozial-emotionale Klima? | (1) Überhaupt nicht gefährlich/(7) Sehr gefährlich |
|  |  | for individual physical health | Für wie gefährlich halten Sie die Art und Weise wie die österreichischen Medien über das Coronavirus/Saisonale Grippe berichten... (4) für die physische Gesundheit des Einzelnen? | (1) Überhaupt nicht gefährlich/(7) Sehr gefährlich |
|  | Usefulness Public Policies | in general | Die österreichischen Regierung hat Maßnahmen vorgenommen, um die weitere Ausbreitung des Coronavirus/Saisonalen Grippevirus einzugrenzen. Wie nützlich sind diese staatlichen Maßnahmen Ihrer Meinung nach...(1) im Allgemeinen? | (1) Überhaupt nicht nützlich/(7) Sehr nützlich |
|  |  | for national economy | Die österreichischen Regierung hat Maßnahmen vorgenommen, um die weitere Ausbreitung des Coronavirus/Saisonalen Grippevirus einzugrenzen. Wie nützlich sind diese staatlichen Maßnahmen Ihrer Meinung nach...(2) für die nationale Wirtschaft? | (1) Überhaupt nicht nützlich/(7) Sehr nützlich |
|  |  | for national social-emotional climate | Die österreichischen Regierung hat Maßnahmen vorgenommen, um die weitere Ausbreitung des Coronavirus/Saisonalen Grippevirus einzugrenzen. Wie nützlich sind diese staatlichen Maßnahmen Ihrer Meinung nach...(3) für das nationale sozial-emotionale Klima? | (1) Überhaupt nicht nützlich/(7) Sehr nützlich |
|  |  | for individual physical health | Die österreichischen Regierung hat Maßnahmen vorgenommen, um die weitere Ausbreitung des Coronavirus/Saisonalen Grippevirus einzugrenzen. Wie nützlich sind diese staatlichen Maßnahmen Ihrer Meinung nach...(4) für die physische Gesundheit des Einzelnen? | (1) Überhaupt nicht nützlich/(7) Sehr nützlich |
|  | Usefulness Media Communication | in general | Für wie nützlich halten Sie die Art und Weise wie die österreichischen Medien über das Coronavirus/Saisonalen Grippevirus berichten... (1) im Allgemeinen? | (1) Überhaupt nicht nützlich/(7) Sehr nützlich |
|  |  | for national economy | Für wie nützlich halten Sie die Art und Weise wie die österreichischen Medien über das Coronavirus/Saisonalen Grippevirus berichten... (2) für die nationale Wirtschaft? | (1) Überhaupt nicht nützlich/(7) Sehr nützlich |
|  |  | for national social-emotional climate | Für wie nützlich halten Sie die Art und Weise wie die österreichischen Medien über das Coronavirus/Saisonalen Grippevirus berichten... (3) für das nationale sozial-emotionale Klima? | (1) Überhaupt nicht nützlich/(7) Sehr nützlich |
|  |  | for individual physical health | Für wie nützlich halten Sie die Art und Weise wie die österreichischen Medien über das Coronavirus/Saisonalen Grippevirus berichten... (4) für die physische Gesundheit des Einzelnen? | (1) Überhaupt nicht nützlich/(7) Sehr nützlich |
|  | Knowledge | for public policies | Wie viel wissen Sie Ihrer Meinung nach über die von der österreichischen Regierung vorgenommenen Maßnahmen zur Eingrenzung der Ausbreitung des Coronavirus/Saisonalen Grippevirus? (1) | (1) Überhaupt nichts/(7) Sehr viel |
|  |  | for behavior | Wie viel wissen Sie Ihrer Meinung nach über Verhaltensweisen zur Eingrenzung der Ausbreitung des Coronavirus/Saisonalen Grippevirus? (2) | (1) Überhaupt nichts/(7) Sehr viel |
|  |  | in general | Wie viel wissen Sie Ihrer Meinung nach über das Coronavirus/Saisonalen Grippevirus? (3) | (1) Überhaupt nichts/(7) Sehr viel |
|  | Manipulation Check |  | Bitte geben Sie unten in der Liste an, zu welcher Viruserkrankung Sie Ihrer Meinung nach befragt wurden: | (1) Coronavirus/(2) Saisonale Grippe/(3) Masern /(4) Keine dieser Optionen |
| Italy | Emotional Reaction | disease | Quando penso al coronavirus/influenza stagionale mi sento…. | (0) Per niente preoccupato/(10) Estremamente preoccupato |
|  |  | public policies | Quando penso alle politiche pubbliche che il Governo italiano ha emanato per limitare la diffusione del coronavirus/influenza stagionale mi sento... | (0) Per niente preoccupato/(10) Estremamente preoccupato |
|  |  | media | Quando penso al modo in cui i media italiani (giornali, siti giornalistici, TG) parlano del coronavirus/influenza stagionale mi sento... | (0) Per niente preoccupato/(10) Estremamente preoccupato |
|  | Likelihood to get infected |  | Qual è la probabilità che tu venga contagiato dal coronavirus/influenza stagionale nel prossimo mese? | (1) Estremamente bassa/(7) Estremamente alta |
|  | Severity of the disease |  | Quanto è pericoloso il coronavirus/l’influenza stagionale? | (1) Per niente pericoloso/(7) Estremamente pericoloso |
|  | Behavior Capability |  | Per me rispettare le indicazioni comportamentali nel prossimo mese sarà impossibile/possibile | (1) Impossibile/(7) Possibile |
|  | Behavior Control |  | Dipende più che altro da me rispettare le indicazioni comportamentali nel prossimo mese. | (1) Non sono per niente d’accordo/(6) Sono pienamente d’accordo |
|  | Behavior |  | Per piacere indica con quanta frequenza ti impegni a rispettare le indicazioni comportamentali usando una scala da 1 a 7, dove 1 indica "Mai" e 7 indica "Sempre". | (1) Mai/(7) Sempre |
|  | Danger Public Policies | in general | Il Governo italiano ha emanato delle politiche pubbliche per limitare la diffusione del coronavirus/influenza stagionale. Secondo te, quanto queste politiche pubbliche sono pericolose… (1) in generale? | (1) Per niente pericoloso/(7) Estremamente pericoloso |
|  |  | for national economy | Il Governo italiano ha emanato delle politiche pubbliche per limitare la diffusione del coronavirus/influenza stagionale. Secondo te, quanto queste politiche pubbliche sono pericolose…(2) per l’economia nazionale? | (1) Per niente pericoloso/(7) Estremamente pericoloso |
|  |  | for national social-emotional climate | Il Governo italiano ha emanato delle politiche pubbliche per limitare la diffusione del coronavirus/influenza stagionale. Secondo te, quanto queste politiche pubbliche sono pericolose…(3) per il clima socio-emotivo nazionale? | (1) Per niente pericoloso/(7) Estremamente pericoloso |
|  |  | for individual physical health | Il Governo italiano ha emanato delle politiche pubbliche per limitare la diffusione del coronavirus/influenza stagionale. Secondo te, quanto queste politiche pubbliche sono pericolose…(4) per la salute fisica di ogni individuo? | (1) Per niente pericoloso/(7) Estremamente pericoloso |
|  | Danger Media Communication | in general | Quanto percepisci il modo in cui i media italiani parlano del coronavirus/influenza stagionale come pericoloso…(1) in generale | (1) Per niente pericoloso/(7) Estremamente pericoloso |
|  |  | for national economy | Quanto percepisci il modo in cui i media italiani parlano del coronavirus/influenza stagionale come pericoloso…(2) per l’economia nazionale | (1) Per niente pericoloso/(7) Estremamente pericoloso |
|  |  | for national social-emotional climate | Quanto percepisci il modo in cui i media italiani parlano del coronavirus/influenza stagionale come pericoloso…(3) per il clima socio-emotivo nazionale? | (1) Per niente pericoloso/(7) Estremamente pericoloso |
|  |  | for individual physical health | Quanto percepisci il modo in cui i media italiani parlano del coronavirus/influenza stagionale come pericoloso…(4) per la salute fisica di ogni individuo? | (1) Per niente pericoloso/(7) Estremamente pericoloso |
|  | Usefulness Public Policies | in general | Il Governo italiano ha emanato delle politiche pubbliche per limitare la diffusione del coronavirus/influenza stagionale. Secondo te, quanto queste politiche pubbliche sono utili…(1) in generale? | (1) Per niente utili/(7) Estremamente  utili |
|  |  | for national economy | Il Governo italiano ha emanato delle politiche pubbliche per limitare la diffusione del coronavirus/influenza stagionale. Secondo te, quanto queste politiche pubbliche sono utili…(2) per l’economia nazionale? | (1) Per niente utili/(7) Estremamente  utili |
|  |  | for national social-emotional climate | Il Governo italiano ha emanato delle politiche pubbliche per limitare la diffusione del coronavirus/influenza stagionale. Secondo te, quanto queste politiche pubbliche sono utili…(3) per il clima socio-emotivo nazionale? | (1) Per niente utili/(7) Estremamente  utili |
|  |  | for individual physical health | Il Governo italiano ha emanato delle politiche pubbliche per limitare la diffusione del coronavirus/influenza stagionale. Secondo te, quanto queste politiche pubbliche sono utili…(4) per la salute fisica di ogni individuo? | (1) Per niente utili/(7) Estremamente  utili |
|  | Usefulness Media Communication | in general | Quanto percepisci il modo in cui i media italiani parlano del coronavirus/influenza stagionale come utile…(1) in generale? | (1) Per niente utili/(7) Estremamente  utili |
|  |  | for national economy | Quanto percepisci il modo in cui i media italiani parlano del coronavirus/influenza stagionale come utile…(2) per l’economia nazionale? | (1) Per niente utili/(7) Estremamente  utili |
|  |  | for national social-emotional climate | Quanto percepisci il modo in cui i media italiani parlano del coronavirus/influenza stagionale come utile…(3) per il clima socio-emotivo nazionale? | (1) Per niente utili/(7) Estremamente  utili |
|  |  | for individual physical health | Quanto percepisci il modo in cui i media italiani parlano del coronavirus/influenza stagionale come utile…(4) per la salute fisica di ogni individuo? | (1) Per niente utili/(7) Estremamente  utili |
|  | Knowledge | for public policies | Quanto pensi di sapere sulle politiche pubbliche emanate dal Governo italiano per limitare la diffusione del coronavirus/influenza stagionale? (1) | (1) Non so assolutamente nulla a riguardo/(7) So tutto a riguardo |
|  |  | for behavior | Quanto pensi di sapere sulle indicazioni comportamentali emanate dal Governo italiano per limitare la diffusione del coronavirus/influenza stagionale? (2) | (1) Non so assolutamente nulla a riguardo/(7) So tutto a riguardo |
|  |  | in general | Quanto pensi di sapere sul coronavirus/influenza stagionale? (3) | (1) Non so assolutamente nulla a riguardo/(7) So tutto a riguardo |
|  | Manipulation Check |  | Per piacere, tra quelle proposte nella lista qui sotto, indica rispetto a quale malattia virale ti è stato chiesto di dare la tua opinione: | (1) Coronavirus/(2) Influenza stagionale/(3) Morbillo/(4) Nessuna di queste |

**Section 3. Additional analysis**

***3.1 Means and standard deviations of the main variables in the three countries***

*Table S4. Means and standard deviations of main variables by Country.*

|  | UK |  | Austria |  | Italy |
| --- | --- | --- | --- | --- | --- |
|  | M (SD) |  | M (SD) |  | M (SD) |
| Perceived threat^***^ | 3.96_H_ (1.28) |  | 3.50_L_ (1.11) |  | 3.42_L_ (1.18) |
| Worry^***^ | 4.25_H_ (1.80) |  | 3.17_L_ (1.69) |  | 3.15_L_ (1.83) |
| Capability^**^ | 6.53_L_ (0.86) |  | 6.72_H_ (0.65) |  | 6.75_H_ (0.57) |
| Control^***^ | 5.28_L_ (1.21) |  | 5.60_H_ (0.75) |  | 5.64_H_ (0.70) |
| Knowledge^*^ | 4.88_H-L_ (1.30) |  | 5.05_H_ (1.25) |  | 4.68_L_ (1.27) |
| Danger public policies ^**^ | 3.80_H_ (1.48) |  | 3.90_H_ (1.39) |  | 3.37_L_ (1.59) |
| Usefulness public policies^***^ | 4.05_L_ (1.31) |  | 4.01_L_ (1.21) |  | 4.55_H_ (1.11) |
| Danger media communication | 4.31 (1.65) |  | 4.26 (1.65) |  | 4.03 (1.82) |
| Usefulness media communication^***^ | 3.80_H_ (1.53) |  | 3.77_H_ (1.32) |  | 3.27_L_ (1.42) |
| TEIque^***^ | 4.89_L_ (0.76) |  | 5.25_H_ (0.68) |  | 4.85_L_ (0.67) |
| Belief in conspiracy theories^***^ | 2.67_H_ (0.86) |  | 2.30_M_ (0.85) |  | 2.08_L_ (0.72) |
| Trust in science^***^ | 3.83_M_ (0.74) |  | 3.60_L_ (0.63) |  | 4.15_H_ (0.67) |
| Trust in politics^*^ | 5.54_L_ (1.44) |  | 5.78_H-L_ (1.51) |  | 5.92_H_ (1.55) |
| Trust in media | 3.71 (1.14) |  | 3.72 (1.20) |  | 3.61 (1.10) |

Note: Results of the post-hoc (Scheffè) test are indicated by subscript (H= higher mean, L= lower mean, M= medium mean). If two countries present the same letter their means for that specific variable were not significantly different. ““H-L” is used to indicate that a mean was not significantly different than the higher (H) and the lower (L) mean. Significant contrasts were reported with asterisks as superscripts to the variables’ labels ^*^p-value < 0.05, ^**^ p-value < 0.01, ^***^ p-value < 0.001

***3.2 Analysis of the interaction of Country and Viral Disease on Worry***

*Table S5. Mean and Standard Deviations of the Worry variable by Country and Viral Disease.*

| Viral Disease | Country | Mean | SD | N |
| --- | --- | --- | --- | --- |
| Seasonal flu | UK | 28.199 | 157.893 | 87 |
|  | Austria | 21.159 | 151.553 | 69 |
|  | Italy | 16.842 | 119.368 | 76 |
| Coronavirus | UK | 52.700 | 111.747 | 121 |
|  | Austria | 38.750 | 142.196 | 104 |
|  | Italy | 43.926 | 127.941 | 90 |

*Table S6. Scheffe post hoc results of the interaction of Country and Viral Disease on Worry.*

| Comparisons | Mean difference | *p* | LCL | UCL |
| --- | --- | --- | --- | --- |
| AU Seasonal flu - AU Coronavirus | -1.759 | < .001 | -2.457 | -1-61 |
| AU Seasonal flu - IT Seasonal flu | 0.432 | .591 | -0.316 | 1.180 |
| AU Seasonal flu - IT Coronavirus | -2.277 | < .001 | -2.996 | -1.557 |
| AU Seasonal flu - UK Seasonal flu | -0.704 | .064 | -1.430 | 0.021 |
| AU Seasonal flu - UK Coronavirus | -3.154 | < .001 | -3.832 | -2.475 |
| AU Coronavirus - IT Seasonal flu | -2.191 | < .001 | 1.151 | 2.870 |
| AU Coronavirus - IT Coronavirus | -0.517 | 0.213 | -1.165 | 0.130 |
| AU Coronavirus - UK Seasonal flu | 1.055 | < .001 | 0.401 | 1.708 |
| AU Coronavirus - UK Coronavirus | -1.395 | < .001 | -1.996 | -0.793 |
| IT Seasonal flu - IT Coronavirus | -2.708 | < .001 | -3.409 | -2.008 |
| IT Seasonal flu - UK Seasonal flu | -1.136 | < .001 | -1.842 | -0.430 |
| IT Seasonal flu - UK Coronavirus | -3.586 | < .001 | -4.244 | -2.297 |
| IT Coronavirus - UK Seasonal flu | 1.573 | < .001 | 0.896 | 2.249 |
| IT Coronavirus - UK Coronavirus | -0.877 | < .001 | -1.503 | -0.251 |
| UK Seasonal flu - UK Coronavirus | -2.450 | < .001 | -3.082 | -1.818 |

***Section 3.3 Additional path model tested.***

We tested the effect of trait emotional intelligence, which has been found to be related to preventive actions, emotional reactions, and risk perception in health-related models (Fernández-Abascal & Martín-Díaz, 2015; Mikolajczak et al., 2015; Peña-Sarrionandia et al., 2015; Sevdalis et al., 2007). The results of a model investigating the effect of trait emotional intelligence on Worry, Perceived threat and Behavior are shown in Path Model 3.

The model had a moderately good model fit, χ^2^ (4, N = 547) = 9.43, *p* = .051, RMSEA = .050, p= .434, the CFI = .990, BIC= 7800.0, and the addition of the TEIque did not modify the relationships between the variables shown in the previous model. Our findings illustrate that participants with higher trait emotional intelligence also reported lower Worry but higher engagement in self-protective behaviors. Moreover, higher emotional intelligence was associated, through its effects on Worry, with lower Perceived threat, *z* = -3.44*, p* = .001*,* 95% CI [-.18, -.05]. In addition to the direct positive effect on Behavior, results also revealed that higher emotional intelligence had a marginally significant negative indirect effect (by its relationship with lower Worry and Perceived threat, *z* = -1.77*, p* = .077*,* 95% CI [-.03, .002]. Thus, the direct and indirect effects of emotional intelligence on self-protective behaviors are in opposite directions.

Moreover, in line with the literature on the role of Knowledge in risk perception (Siegrist & Cvetkovich, 2000), we tested the effect of Knowledge on the model as a mediator on the effect of Viral Disease on Behavior (i.e., Path Model 4). The model is significantly worse than the fully specified model, χ^2^ (8, N = 547) = 65.56, p < .001, RMSEA = .11, *p* < .001, the CFI = .924, BIC= 9473.0, and significantly worse than Path Model 2 (
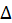
 χ^2^ (4) = 58.69, *p* < 0.01). However, the analysis provided some noteworthy insights. Our participants report to know more about the coronavirus than the season flu, *z* = 12.22*, p* < 0.01*,* 95% CI [1.01, 1.39]. The effect of Viral Disease on Behavior, considering Knowledge as one of the mediators, was significant, *z* = 6.13*, p* < 0.01*,* 95% CI [.23, .45]. Thus, being in the coronavirus (vs seasonal flu) condition made participants perceive that they have higher knowledge and this was associated with higher self-reported engagement in self-protective behaviors.


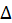


Finally, we tested the expected effect of Control and Capability on Behavior. After controlling for the effect of Control and Capability on Behavior (i.e., Path Model 5) the relationship between our main variables (Path Model 2) remained invariant and Capability (*z* = 8.51*, p* < 0.01*,* 95% CI [.37, .60]) but not Control (*z* = 0.76*, p* = .446*,* 95% CI [-.05, .12]) showed a significant positive effect on the Behavior.

***Section 3.4 Fit of the main model separately for the three countries.***

We also fitted our main model (i.e., Path Model 2) separately for the UK (χ^2^ (4, N = 208) = 2.90, *p* = .574, RMSEA < .001, *p* = .77, CFI = 1.00, BIC= 2527.4), the Austrian (χ^2^ (4, N = 173) = 6.48, *p* = .166, RMSEA = .06, *p* = .34, CFI = .976, BIC= 2142.9) and the Italian (χ^2^ (4, N = 166) = 6.20, *p* = .185, RMSEA= .058, *p* = .36, the CFI = .990, BIC= 1927.5) samples. In the UK sample, Frame and Viral Disease had no indirect effect on Behavior. However, the Viral Disease had an indirect effect on Perceived threat and Worry had an indirect effect on Behavior. Thus, participants in the coronavirus (vs seasonal flu) condition were more worried and this led to higher Perceived threat. Higher Worry was also associated with higher Perceived threat and this led to higher engagement on self-protective behaviors. The Austrian sample showed the same significant patterns as the UK sample. Finally, in the Italian sample, the indirect effect of Worry on Behavior and the indirect effect of Viral Disease on Perceived threat were significant and similar to the results found in the Austrian and UK sample. However, in Italy, Viral Disease had a significant indirect effect on Behavior and the Frame had an indirect effect on Perceived threat (not significant in Austria and UK). The relationship between the variables is similar to the one reported in the main model (Figure 1 in the paper).
